# Micronuclei to detect *in vivo* chemotherapy damage in a p53 mutated solid tumour

**DOI:** 10.1038/sj.bjc.6601163

**Published:** 2003-08-12

**Authors:** G Driessens, L Harsan, B Robaye, D Waroquier, P Browaeys, X Giannakopoulos, T Velu, C Bruyns

**Affiliations:** 1 Interdisciplinary Research Institute (IRIBHM), Faculty of Medicine, Université Libre de Bruxelles, 1070 Brussels, Belgium; 2 Faculty of Physics, Babes-Bolyai University, 3400 Cluj-Napoca, Rumania; 3 Department of Medical Oncology, Erasme Hospital, 1070 Brussels, Belgium

**Keywords:** micronuclei, apoptosis, p53, *γ*-irradiation, chemotherapy, glioma

## Abstract

Apoptosis induction and micronuclei formation were compared following cytotoxic treatments in two rat glioma differing in p53 integrity. *In vitro*, micronuclei emergence but not apoptosis was linked to the p53 mutated status. *In vivo*, micronuclei assays were more sensitive to evaluate DNA damage induced by chemotherapy in a p53-mutated solid tumour.

Most anticancer agents exert their action by triggering apoptosis (Mow *et al*, 2001). The product of the tumour-suppressor gene p53 is a key mediator in this process (Soussi, 2000). In normal cells, wild-type p53 either arrests cell proliferation until the genetic damage is repaired or forces the cell to commit apoptosis (Cadwell and Zambetti, 2001). In cancer cells, where p53 alleles are often mutated, decrease in apoptosis can be compensated by the process of mitotic catastrophe, a form of cell death resulting from abnormal mitosis and leading to the formation of cells with multiple micronuclei (MN) (Roninson *et al*, 2001). This study aimed to compare induction of apoptosis and MN formation after chemo- or radiotherapy, *in vitro* in two rat glioma cell lines differing in p53 integrity, the 9L expressing a mutated p53 gene and the C6 the wild-type gene, and *in vivo* on established 9L solid tumours.

## MATERIALS AND METHODS

### Animals and cell lines

All experiments on Fischer 344 rats have been carried out with local ethical committee approval and met the standards required by the UKCCCR guidelines (Workman *et al*, 1998). The 9L gliosarcoma cell line and the C6 glioblastoma cell line were maintained in complete medium, RPMI 1640 supplemented with 10% fœtal calf serum, 1% L-glutamine, 1% sodium pyruvate, 1% nonessential amino acids, 100 IU ml^−1^ penicillin and 100 *μ*g ml^−1^ streptomycin.

### *In vitro* and *in vivo* radio- or chemotherapy

For *in vitro* treatments, 9L and C6 cells were *γ*-irradiated (80 Gy, ^137^Cs irradiator) or treated for 2 h with chemotherapeutic drugs and recultured during 24 or 72 h for apoptosis tests or 4 h for MN assays. For *in vivo* treatments, tumour-bearing rats (10^5^ 9L s.c. at day 0) received i.p. injections of cisplatin (1 mg kg^−1^), or were irradiated locally at the tumour site (20 Gy) at days 4, 11 and 18 and were killed the day after. The tumours were then dissected and prepared distinctly for apoptosis or MN assays.

### Apoptosis assays

#### Analysis of caspase 3 activity

Caspase-3 activation was measured *in vitro* by the cleavage of a specific fluorogenic substrate (Ac-DEVD-AMC, BD Biosciences, Erembodegem, Belgium). Briefly, 24 or 72 h after treatment, cells were lysed and incubated first in protease buffer and then for 2 h at 37°C with Ac-DEVD-AMC substrate (1 mg ml^−1^). The enzyme-catalysed release of fluorescent AMC was measured, by referring to a standard curve, with a fluorimeter, at 380 nm excitation and 440 nm emission wavelengths.

#### Measurement of phosphatidylserine translocation using AnnexinV–FITC

Phosphatidylserine outer translocation on treated cells was detected by flow cytometry (BD FACScan and CellQuest software) using AnnexinV–FITC binding (1 *μ*g ml^−1^) (BD Biosciences) and PI (2 *μ*g ml^−1^) counterstaining.

#### TUNEL assay

Treated tumours excised from rats were directly cryo-preserved in OCT, cut into 10-μm-thick sections and kept at −20°C. Paraformaldehyde 4% fixed tissue sections were permeabilised in PBS–Triton X-100 1% solution and incubated for 1 h at 37°C with TdT enzyme (*In Situ* Cell Death Detection, Roche Molecular Biochemicals, Brussels, Belgium) able to add fluorescein-conjugated nucleotides to the free 3′ ends of DNA fragments generated in apoptotic cells. Slides were analysed under fluorescence microscopy.

#### Micronucleus assay

Micronuclei (MN) are detected in cells that have completed nuclear division following a method developed by Fenech (2000) that identifies such cells by their binucleate (BN) appearance after blocking cytokinesis with cytochalasin-B. Briefly, *in vitro* treated 9L or C6 cells were incubated with cytochalasin-B (3 *μ*g ml^−1^) for 24 h. *Ex vivo* resected treated 9L tumours were first dissociated in DNAse/collagenase solution and recultured before cytochalasin-B was added. After a hypotonic shock in 0.075 M KCl, cells were stained with Hoechst 33342 (2 *μ*g ml^−1^) and examined with a fluorescence microscope, under UV light. BN cells with MN were counted within two independent experiments involving at least triplicates of 500 cells.

#### Statistics

Statistical analysis was done using the unpaired *t*-test.

## RESULTS AND DISCUSSION

**Figure 1:**
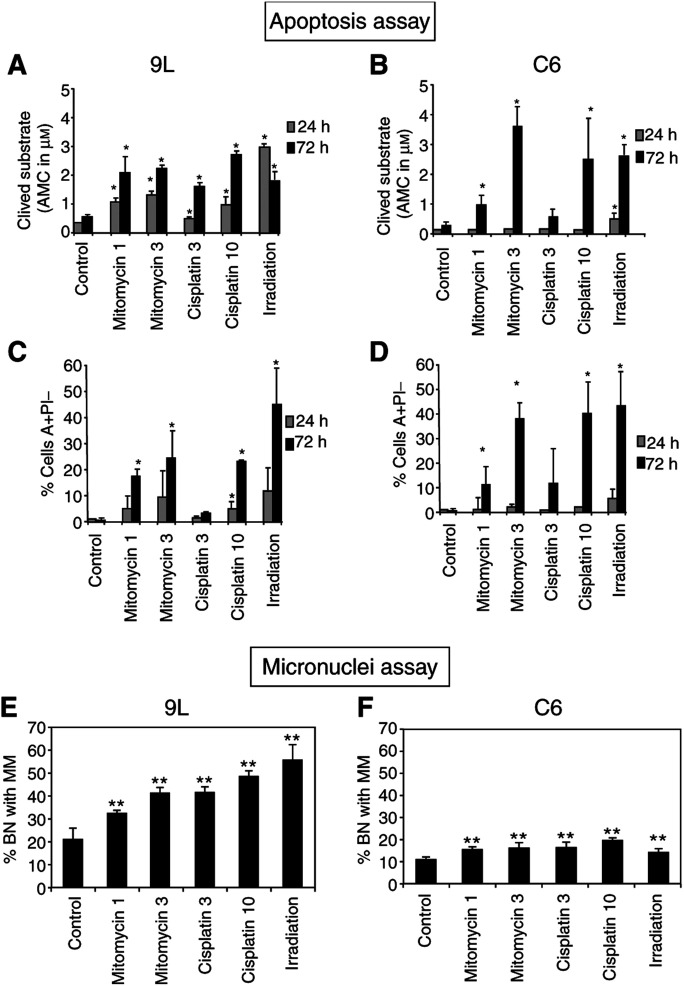
*In vitro* induction of apoptosis or MN formation after chemo- or radiotherapy. Measure of caspase-3 activity in 9L (**A**) and C6 (**C**) cells. Measure of PS externalisation by AnnexinV–FITC binding assay on 9L (**B**) and C6 cells (**D**). Total percentages of 9L (**E**) and C6 (**F**) binucleated cells with MN. Significantly different from the control: ^*^P<0.05, ^**^P<0.01.

We have investigated and compared *in vitro* apoptosis induction and MN formation in p53 mutated 9L or p53 wild-type C6 cells treated either by irradiation or by chemotherapeutic agents (chosen according to their opposite efficacies on tumour cell viability, data not shown). Apoptosis was quantified by relevant tests addressing different stages of the process: measure of caspase-3 activity (Figure 1A and C

) and externalisation of phosphatidylserines (AnnexinV–FITC/PI assay) (Figure 1B and D). Results show, in general, similar induction of apoptosis in 9L or C6 cells. Irradiation and mitomycin C at 3 *μ*g ml^−1^ were the best inducers in both the cell lines and cisplatin at 3 *μ*g ml^−1^ quite less effective. Our results are in agreement with the model in which p53 ‘senses’ DNA damage through direct interaction withDNA-damage sites, and then triggers downstream events leading to apoptosis if the damages are not repaired (Bennett, 1999). Especially, in C6 cells expressing the wild-type p53 protein, hardly any apoptosis was observed after 24 h, increasing with drug concentration and with time. In 9L cells expressing a mutated form of p53, alternative p53-independent pathways of apoptosis could be active, implicating proteins such as p73 (Catani *et al*, 2002).

On the other hand, MN assays were more discriminatory to detect DNA damage in relation to the p53 status of the tumour cells. Indeed, emergence of BN cells with MN was quite high in 9L cells with a mutated p53 gene (Figure 1E) and quite low in C6 cells expressing the wild-type p53 (Figure 1F). Cisplatin at 10 *μ*g ml^−1^ and *γ*-irradiation provoked even such damage in 9L cells that four, five or six MN were often detected per cell, which is unusual (data not shown). On the contrary, in C6 cells, more than three MN per cell were observed rarely (data not shown). The emergence of MN is attributed to the ability of cytotoxic agents to provoke DNA strand breaks either directly as for irradiation or indirectly as the results of drug insertion into the DNA helix (Brabec, 2002). Our data fit with previous observations showing the importance of wild-type p53 protein expression in the balance between cell cycle arrest/DNA repair and apoptosis induction (Ferreira *et al*, 1999). Indeed, very few MN were detectable in C6 cells. On the contrary, in p53-mutated 9L cells, there is less growth arrest or DNA repair and therefore a higher emergence of MN, as described for head and neck carcinoma cells transfected with a mutant p53 (Masunaga *et al*, 2002).

**Figure 2:**
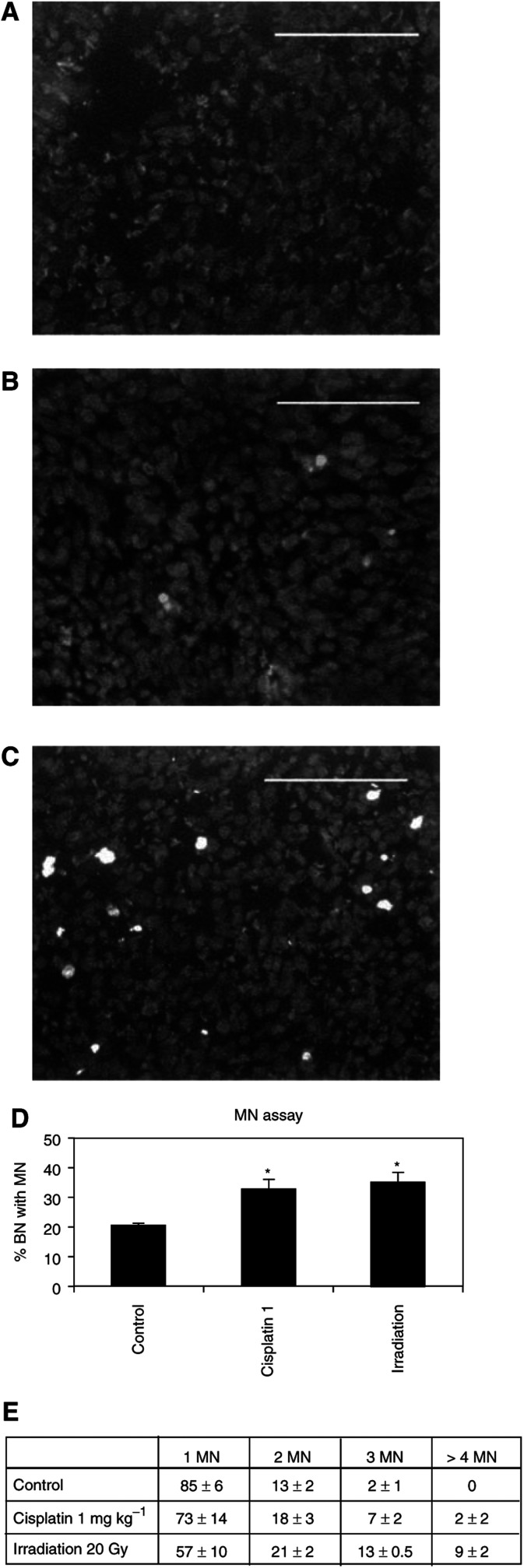
*In vivo* induction of apoptosis or MN formation in 9L tumours after local *γ*-irradiation or systemic injection of cisplatin. TUNEL assay on tumour section from control nontreated rat (**A**), cisplatin (1 mg/kg^−1^) treated rat (**B**) or locally *γ*-irradiated rat (**C**). Total percentages of binucleated (BN) cells with MN (**D**) in 9L tumours. Percentages of BN cells with MN divided into subcategories depending on the number of MN per cell (**E**). ^*^Significantly different from the control (*P*<0.05). Magnification bars=0.1 mm.

*In vivo* induction of apoptosis in established 9L tumours was assessed after local tumour irradiation (20 Gy) or systemic injection of a nontoxic dose of cisplatin (1 mg kg^−1^) (Burger *et al*, 2002). Data from TUNEL assays (Figure 2A–C

) and from anti-active caspase-3 immunostaining (data not shown) revealed the presence of apoptotic cells only after irradiation but not after cisplatin treatment. However, by the MN assay, DNA damages were detected in the same *in vivo* treated 9L tumours also after chemotherapy. Indeed, as shown in Figure 2D, similar emergence of total BN cells with MN was observed for both treatments, even if the number of BN cells bearing ⩾3 MN per cell was increased in the case of *γ*-irradiation (Figure 2E). This result contrasts with the conclusion of apoptosis tests. However, due to the *in vivo* clearance of apoptotic cells by macrophages, there was no means to quantify the time-dependent accumulation of apoptotic cells (Savill and Fadok, 2000).

The MN assay is generally used to determine the *in vivo* genotoxicity of carcinogens in normal cells like human hymphocytes (Gonzalez Borroto *et al*, 2001; Migliore *et al*, 2002). Only a few recent publications reported MN formation in cell lines derived from tumours (Truter *et al*, 2002). We have thus successfully applied this technique on a solid tumour, to evaluate its *in vivo* sensitivity to chemotherapy, assessing the real effect of the drug on the tumour mass rather than a systemic toxicity. Correlating apoptosis induction and MN formation could help to set up new promising strategies of immunotherapy, combining dendritic cell vaccination with radio- or chemotherapy as producing sources of tumour antigens.
